# When Mothers and Fathers Are ‘Gone’: Predicting Intergenerational Cycles of Risk in Opioid-Involved Parents

**DOI:** 10.3390/children12111496

**Published:** 2025-11-04

**Authors:** Maria Khan, Kimberly Renk

**Affiliations:** Department of Psychology, University of Central Florida, 4000 Central Florida Blvd., Orlando, FL 32816, USA

**Keywords:** adverse childhood experiences, attachment, opioid use, mothers, fathers

## Abstract

****Highlights**:**

**What are the main findings?**

- The opioid epidemic and adverse childhood experiences (ACEs) are two major concerns for child welfare systems in the United States of America.
- This study examined a high-risk sample of 101 parents (75 mothers and 26 fathers) who were opioid-involved, who had a child in the 0- to 5-year age range, and who were receiving medication-assisted treatment in the United States of America.

**What are the implications of the main findings?**

- Mothers’ ACEs significantly predicted their ratings of disorganized attachment patterns with their young children, with depression and trauma symptoms explaining greater variance than ACEs alone.
- Fathers’ ACEs were not predictive of insecure/disorganized attachment patterns with their young children.

**Abstract:**

**Background/Objectives:** The opioid epidemic and adverse childhood experiences (ACEs) are two major concerns for child welfare systems. Little is known, however, regarding the mechanisms that perpetuate intergenerational cycles of ACEs and insecure/disorganized parent–young child attachment patterns in the context of parents’ opioid use. **Methods:** A high-risk sample of 101 parents (75 mothers and 26 fathers) who were opioid-involved, who had a child in the 0- to 5-year age range, and who were receiving medication-assisted treatment in the United States of America participated in this study. Parents were mostly White/Caucasian and single. Direct pathways between parents’ reported ACEs and their perceived parent–young child attachment patterns, as well as indirect pathways through substance use severity, depression, and trauma symptoms, were examined. **Results:** PROCESS analyses suggested that mothers’ ACEs significantly predicted their ratings of disorganized attachment patterns with their young children, with depression and trauma symptoms explaining greater variance than ACEs alone. The results indicated that fathers’ ACEs were not predictive of insecure/disorganized attachment patterns with their young children. Substance use severity was not predictive of parent–child attachment patterns for mothers or fathers. **Conclusions:** Generally, these findings highlighted different patterns among mothers and fathers’ ACEs, their ratings of parent–young child insecure/disorganized attachment, and their psychological sequelae. These findings further suggested the utility of trauma- and attachment-informed parenting interventions for high-risk mothers and fathers in breaking intergenerational cycles of risk.

## 1. Introduction

The opioid epidemic has garnered growing concern (e.g., [1,2,3]), particularly in child welfare systems in the United States of America. In fact, rates of opioid prescriptions, opioid misuse, and opioid-related overdose deaths have been on the rise since the 1990s [4]. These increased rates prompted professionals in the United States of America to describe opioid use disorder as a public health emergency in 2017 [5]. Given this constellation of opioid-related issues, understanding the pathways of risk for intergenerational cycles of adverse childhood experiences (ACEs), insecure/disorganized attachment patterns between parents and their young children, and substance use in opioid-involved populations has become even more important, especially when planning for appropriate parenting and other interventions. Exposure to parents’ substance use itself is considered an ACE, with 25.6% of participants in the original ACEs Study endorsing that they had been exposed to a parent who was substance-involved [6]. Further, parents’ substance use increases their risk of maltreating children, removal of children from their care, and failure to achieve reunification after a removal has occurred [7,8]. This increased risk is likely related to these parents’ greater parenting stress, decreased attentiveness and engagement, and harsher discipline. Thus, efforts meant to break intergenerational cycles and to prevent maladaptive outcomes for young children necessitate a lens that is both trauma-informed and attachment-focused [9]. Consistently, the goal of this study was to examine potential pathways from ACEs to perceived insecure/disorganized attachment patterns in a sample of mothers and fathers who were opioid-involved.

When examining intergenerational cycles, attachment needs to be considered. Such consideration is particularly important in families with young children, as young children actively form attachments with caregivers. Of particular interest for this study, attachment theory has demonstrated the importance of young children establishing safe and secure internal working models from which they can view their world [10]. Safe and secure internal working models, or mental maps of relationships, are formed when young children are cared for consistently and reliably by caregivers who are warm but firm. When internal working models are not safe and secure, young children (and individuals, in general) display heightened risk of insecure or even disorganized attachment, as well as later emotional regulation difficulties, poor coping skills, and relationship insecurity [11]. ACEs (e.g., abuse, neglect, and household dysfunction), especially at the hands of primary attachment figures, can be highly detrimental to young children’s early internal safety representations [12]. Understanding pathways to insecure/disorganized attachment patterns is important because childhood adversity is far from uncommon and because the majority of children who were maltreated were perpetrated against by a parent [6].

Much of the literature has considered direct consequences stemming from childhood adversity and other ACEs. Infants are predisposed biologically to attach to their caregivers, as caregivers protect [13], foster learning of regulation, and shape brain development via early interaction [14]. Thus, the connection between childhood adversity and insecure attachment is salient. Childhood adversity has been related consistently to insecure attachment for caregivers during their own childhoods [12] and for parents’ own young children during their parenting [15,16]. Further, childhood adversity is related to maladaptive coping mechanisms (e.g., substance use) [6,17] and to psychological sequelae (e.g., depression and trauma symptoms) [18]. Rather than being independent, these parental risk factors tend to coexist [19], with these factors being possible mechanisms of connection between ACEs and perceived insecure/disorganized attachment patterns. Further, these factors need to be considered in the context of life stressors [20].

In fact, research has suggested a significant intergenerational component to childhood adversity [21,22], substance use [23], and attachment [12]. Retrospective and prospective data indicate that these variables increase individuals’ risk of maltreating their own children, using substances themselves, and fostering insecure attachment with their own children. Less is known, however, regarding why such intergenerational cycles continue to repeat, as few studies have investigated the overlapping predictive and indirect relationships needed to understand parent–young child attachment [24]. Thus, this study examined both direct and indirect pathways among ACEs, perceived insecure/disorganized attachment patterns, and psychological sequelae. As such intergenerational cycles have been examined less in fathers than mothers [25], attempts were made to include both mothers and fathers in this study. Overall, it is vital to identify ports of entry for breaking these cycles so that young children do not have to endure the same issues as previous generations.

### The Present Study

Although childhood adversity and other ACEs have been examined widely, the variables noted above have yet to be examined collectively in an interactional model for parents who are opioid-involved (to the authors’ knowledge). Moreover, research has called for further study of the mechanisms by which ACEs and parent–young child attachment patterns may be related [24,26]. These relationships are particularly important for parents who are substance-involved and who consequently exhibit a higher risk of maladaptive parenting and insecure/disorganized attachment [7]. To date, research on parenting, attachment, and parents’ substance use has largely addressed the relationship between young children and their mothers [27], to the exclusion of fathers. Even with increasing rates of opioid misuse in the United States of America [28], especially in males [29], only one review of opioid use in fathers exists [1] to the authors’ knowledge.

To address these gaps in the literature, this study examined mediating factors that may be driving the relationships between parents’ ACEs and their perceptions of parent–young child insecure/disorganized attachment patterns. This study recruited a sample of mothers and fathers who were opioid-involved to examine the direct and indirect relationships between their ACEs and parent–young child attachment patterns using the parents’ substance use severity, depression, and trauma symptoms as mediators in a PROCESS mediational model [30]. It was expected that mothers and fathers’ ACEs would predict their respective ratings of insecure/disorganized attachment patterns with their young children and that substance use severity, depression, and trauma symptoms would serve as mediators in this relationship. It was hoped that information gained from this study would help to better inform parenting programs in facilitating secure attachment between mothers and fathers with their young children when substances are involved.

## 2. Materials and Methods

### 2.1. Participants

A sample of 101 parents (75 mothers and 26 fathers) who were opioid-involved, who had young children from 0 to 5 years of age, and who were receiving medication-assisted treatment services in Orlando, Florida, United States of America, were recruited for this study. Parents were recruited from a local outpatient methadone clinic (76% of mothers; 100% of fathers) and women’s residential substance-treatment facilities (24% of mothers). Parents were mostly independent (i.e., there were only four mother–father dyads who answered about a shared child). Eligibility criteria included receiving medication-assisted treatment (i.e., methadone, suboxone, or vivitrol), being at least 18 years of age, and having active involvement with their young children. Parents responded to study questionnaires about their oldest child between 0 and 5 years of age.

Mothers averaged 33.08 years of age, identified mostly as White/Caucasian (80%), and were mostly single (70.7%). Fathers averaged 34.62 years of age, identified mostly as White/Caucasian (73.1%), and were mostly single (73.1%). The young children for whom parents provided ratings averaged 2.83 years for mothers and 3.14 years for fathers. Less than half of the mothers (42.7%) and fathers (46.2%) reported living with their young child’s other parent. Mothers reported mostly being unemployed (60.3%) and having an average household income of <USD 20,000/year (51.3%). In contrast, fathers reported mostly being employed (84.6%) and having an average household income of <USD 40,000/year (72.9%). About 42.5% of mothers and 30.8% of fathers had completed some college. Regarding lifetime use of nonmedical substances, mothers averaged five substances, and fathers averaged six substances. Opiates were the ‘drug of choice’ for 87.8% of mothers and 60% of fathers. Most mothers (82.4%) and fathers (61.5%) were currently receiving methadone. See Table 1 for complete demographic data.

### 2.2. Procedure

This study was approved by the university’s IRB as well as by the research review committee of the partnering mental health agency. Researchers were available at the local outpatient methadone clinic and women’s residential substance-treatment facilities during times when individuals arrived for their medication-assisted treatment. Individuals were provided with a flyer about the study and were asked if they met the inclusion criteria. Those individuals who met the inclusion criteria and who agreed to participate reviewed a consent form (i.e., no identifying information was collected). Measures (described below) were then administered via a pencil-and-paper survey. Following completion, a debriefing form explaining the purpose of the study, as well as relevant resources for parenting programs, was distributed to participants. As an incentive, participants received a USD 5.00 Walmart gift card for their participation.

### 2.3. Measures

*Demographics*. A brief demographic questionnaire asked about parents’ age, ethnicity, occupation, education, household, and other general characteristics. The questionnaire assessed lifetime use of substances to yield a total number of substances score (as laid out in [31]). The total number of substance scores demonstrated good internal consistency (α = 0.83).

*Adverse Childhood Experiences (ACEs).* The *Adverse Childhood Experiences (ACEs) Questionnaire* [6] assessed parents’ exposure to ACEs in the first 18 years of their lives. Parents endorsed exposure to each item in a *Yes* or *No* format, with *Yes* responses added to yield a Total Exposure score ranging from 0 to 10. The ACEs questionnaire had good internal consistency previously (α = 0.86) [19] and in this study (α = 0.82).

*Parent–Young Child Attachment.* The *Experience in Close Relationships Scale* (ECR) [32] assessed parents’ perceptions of insecure anxious and insecure avoidant attachment with their young children. As this scale was developed for adult attachment, the ECR was adapted for parent–young child attachment. The ECR contains two subscales constructed from 36 items that are rated on a 7-point Likert-type scale ranging from 1 (*Disagree Strongly*) to 7 (*Agree Strongly*). Insecure anxious attachment assessed parents’ perceptions of their young children’s fears of being left, needs for reassurance, and distress when parents are not available. Insecure avoidant attachment assessed parents’ perceptions of their young children’s discomfort with closeness, need for self-reliance, and reluctance to be dependent. The ECR has displayed excellent internal consistency (α = 0.91 for anxious; α = 0.94 for avoidant) in prior studies [32] and displayed excellent internal consistency when adapted for mother–young child attachment (α = 0.88 for anxious; α = 0.94 for avoidant) [19]. In this study, the ECR had good internal consistency (α = 0.87 for anxious; α = 0.87 for avoidant).

The *Caregiving Helplessness Questionnaire* (CHQ) [33] assessed parents’ perceptions of disorganized attachment with their young children. The CHQ consists of 25 items that are rated on a 5-point Likert-type scale ranging from 1 (*Not Characteristic at All*) to 5 (*Very Characteristic*) and that load into three factors (i.e., Mother and Child Frightened, Mother Helpless, and Child Caregiving). The Mother and Child Frightened subscale (which measures mothers feeling frightened by their child or their child feeling frightened by them) and the Mother Helpless subscale (which measures mothers’ feelings of being out of control when with their child) were significantly related to disorganized attachment patterns [34] and, thus, were used in this study. Internal consistency was good for Mother and Child Frightened (α = 0.83) and Mother Helpless (α = 0.89) in a previous study [19]. As the CHQ was designed for mothers, items were reformatted for use with ‘parents.’ Internal consistency for Parent and Child Frightened (later referred to as frightened–disorganized; α = 0.56 for parents altogether; α = 0.53 for mothers and α = 0.60 for fathers independently) and Parent Helpless (later referred to as helpless–disorganized; α = 0.64 for parents altogether and for mothers and fathers independently) was mostly adequate in this study.

*Substance Use Severity.* The *Inventory of Drug Use Consequences* (InDUC) [35] assessed parents’ consequences of lifetime use of substances. The InDUC consists of 50 items rated with a *Yes* or *No* format that yield a Total InDUC score. The InDUC demonstrated excellent internal consistency previously (α = 0.96) [36] and in this study (α = 0.95). The *Minnesota Substance Abuse Problems Scale* (M-SAPS) [37] assessed parents’ lifetime substance use-related problems. The M-SAPS consists of 61 items rated with a *Yes* or *No* format and yields a total score. The total score showed excellent internal consistency (α = 0.94) in this study.

A validated measure is yet to exist for assessing the intensity of parent-specific substance use difficulties. Thus, this study factored out parent-specific items from the InDUC and M-SAPS to create a brief parental *substance use severity* (SUS) scale. The SUS scale assessed the intensity of substance use consequences on caregiving, home, financial responsibilities, and interpersonal difficulties. The SUS contained 19 items rated with a *Yes* or *No* format, with items 1 through 11 originally belonging to the M-SAPS and items 12 through 19 originally belonging to the InDUC. Parents’ *Yes* responses were added to yield a total SUS score. The SUS displayed good internal consistency (α = 0.85) and was used for analyses in this study.

*Depression.* The *Beck Depression Inventory-II* (BDI) [38] assessed parents’ current depression symptoms. The BDI contains 21 items that are rated using a 0 to 3 scale, with higher ratings indicating increased severity of depression. The BDI total score had excellent internal consistency previously (α = 0.92) [38] and in this study (α = 0.93).

*Trauma Symptoms.* The *Impact of Events Scale—Revised* (IES-R) [39] assessed parents’ trauma symptoms as they relate to *DSM-IV* PTSD criteria. The IES-R consists of 22 items rated on a Likert-type scale ranging from 0 (*Not at all*) to 4 (*Extremely*) regarding distress over the past seven days from parents’ experienced trauma (in this case, the most traumatic of their ACEs). The IES-R consists of three subscales (Intrusion, Avoidance, and Hyperarousal), which yield a total stress score. The IES-R had excellent internal consistency for the total score previously (α = 0.96) [39] and in this study (α = 0.96).

## 3. Results

### 3.1. Descriptive Statistics

All data were screened for violations of missing data, normality, outliers, and differences between groups. Despite unequal sample sizes, both mother and father data were found to be distributed normally and to have equal variances. No outliers were identified for either group. Descriptive statistics were examined for each group (see Table 2), and independent-samples *t*-tests examined mean differences between groups (see Table 3).

Mothers and fathers reported overall low ACEs. Nonetheless, 52% of mothers and 42.1% of fathers reported four or more ACEs. No significant differences were found between this sample and another opioid-involved sample undergoing substance treatment [40] on the average ACE score for mothers, *t*(74) = 0.13, *p* < 0.70, or fathers, *t*(25) = −0.64, *p* < 0.24. Mothers reported overall low levels of avoidant, anxious, frightened–disorganized, and helpless–disorganized attachment with their young children. Fathers reported overall low levels of avoidant attachment but moderate levels of anxious attachment and low-to-moderate levels of frightened–disorganized and helpless–disorganized attachment with their young children.

Mothers and fathers reported having a moderate-to-high level of substance-related consequences on the InDUC (*M* = 36.51 and *M* = 39.58, respectively) and M-SAPS (*M* = 40.65 and *M* = 46.35, respectively). These scores exceeded those of the original clinical samples of women and men for the InDUC (i.e., *M* = 34.59 and 37.28, respectively) [35] and of both community and clinical samples for the M-SAPS (i.e., *M* = 28.50 and 34.70, respectively) [41]. On the SUS, mothers and fathers reported high overall parent-related substance use severity.

Mothers scored in the low-to-mild range, and fathers scored in the mild range for depression. Relative to the range of possible scores on the BDI, 58% of mothers and 61% of fathers fell within minimal range, 21% of mothers and 8% of fathers fell within mild range, 15% of mothers and 19% of fathers fell within moderate range, and 7% of mothers and 11% of fathers fell within severe range of depression. Mothers averaged low-to-moderate trauma symptoms, whereas fathers averaged moderate trauma symptoms.

### 3.2. Pearson Correlation Analyses

Multicollinearity was evaluated to verify that the variables of interest were not a cause of biased regression analyses. Next, separate Pearson (two-tailed) correlation analyses were conducted for mothers and fathers. Correlations provided evidence for the relationships among the variables of interest. Due to the number of correlations examined, a Bonferroni correction was considered, resulting in an adjusted *p*-value of 0.0003. See Table 4 and Table 5.

### 3.3. Mediation Analyses

To examine the overall model in predicting direct and indirect pathways from mothers and fathers’ ACEs to their perceptions of parent–young child insecure/disorganized attachment patterns with substance use severity, depression, and trauma symptoms as potential mediators, regression analyses were conducted using PROCESS [30].

#### 3.3.1. First Set of Mediation Analyses: Mothers’ ACEs Predicting Mother–Young Child Attachment Patterns

First, the hypothesized path (i.e., direct effect) from mothers’ ACEs to mother–young child attachment patterns was examined. Contrary to our hypotheses, mothers’ ACEs did not predict significantly avoidant, *F* (1, 71) = 0.004, *b* = 0.003, *t* = 0.06, *p* < 0.95, *R*^2^ < 0.001, or anxious, *F* (1, 71) = 2.50, *b* = 0.07, *t* = 1.58, *p* < 0.12, *R*^2^ = 0.03, attachment patterns. As hypothesized, the paths from mothers’ ACEs to mother–young child frightened–disorganized, *F* (1, 72) = 6.76, *b* = 0.33, *t* = 2.60, *p* < 0.01, *R*^2^ = 0.09, and helpless–disorganized, *F* (1, 72) = 6.45, *b* = 0.39, *t* = 2.54, *p* < 0.01, *R*^2^ = 0.08, attachment patterns were positive and significant. Given this pattern of results, only the disorganized attachment variables were examined further in the overall model.

#### 3.3.2. Mothers’ ACEs Predicting Substance Use Severity, Depression, and Trauma Symptoms

Contrary to our hypotheses, mothers’ ACEs did not predict significantly substance use severity (via the SUS), *F* (1, 72) = 7.5, *b* = 0.12, *t* = 0.87, *p* < 0.39, *R*^2^ = 01, or depression, *F* (1, 72) = 1.65, *b* = 0.52, *t* = 1.28, *p* < 0.20, *R*^2^ = 0.02. In support of the hypotheses, mothers’ ACEs positively and significantly predicted their trauma symptoms, *F* (1, 72) = 25.65, *b* = 4.17, *t* = 5.06, *p* < 0.001, *R*^2^ = 0.26, such that greater ACEs predicted mothers’ greater trauma symptoms.

#### 3.3.3. Mothers’ Substance Use Severity, Depression, and Trauma Symptoms Predicting Frightened–Disorganized Attachment Patterns

Contrary to our hypotheses, mothers’ substance use severity, *F* (1, 72) = 0.69, *b* = −0.09, *t* = −0.83, *p* < 0.41, *R*^2^ = 0.009, and depression, *F* (1, 72) = 1.89, *b* = 0.05, *t* = 1.37, *p* < 0.17, *R*^2^ = 0.03, did not significantly predict frightened–disorganized attachment patterns. As hypothesized, mothers’ trauma symptoms positively and significantly predicted frightened–disorganized attachment patterns, *F* (1, 72) = 6.45, *b* = 0.39, *t* = 2.54, *p* < 0.01, *R*^2^ = 0.08.

#### 3.3.4. Mothers’ ACEs, Substance Use Severity, Depression, and Trauma Symptoms Predicting Frightened–Disorganized Attachment Patterns

**Figure 1:**
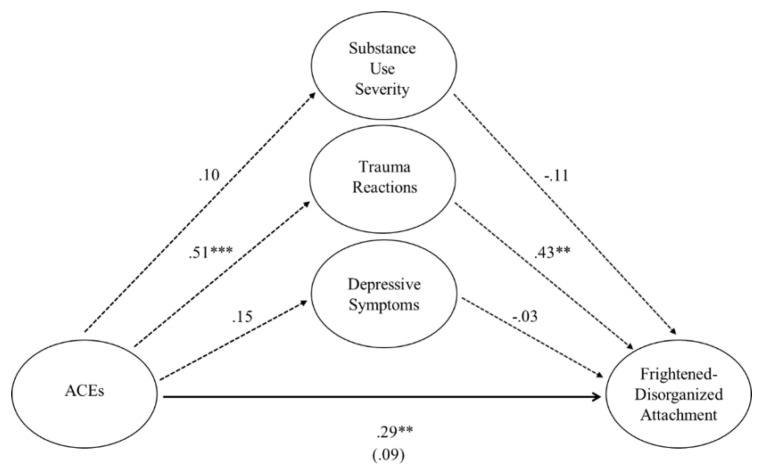
**Standardized regression coefficients for the relationship between mothers’ adverse childhood experiences and frightened–disorganized attachment patterns*****. Note.*** The standardized regression coefficient between mothers’ ACEs and frightened–disorganized attachment patterns, controlling for substance use severity, depression, and trauma, is in parentheses. The bold line indicates a direct path, whereas the dotted lines indicate indirect paths. Significance indicated by ** *p* < 0.01 and *** *p* < 0.001.

Collectively, when all predictor variables were entered into the regression equation to predict mother–child frightened–disorganized attachment patterns, the model was significant, *F* (4, 69) = 5.09, *p* < 0.001, *R*^2^ = 0.23. When mothers’ substance use severity, depression, and trauma symptoms were entered into the equation, mothers’ ACEs no longer significantly predicted frightened–disorganized attachment patterns, *b* = 0.10, *t* = 0.73, *p* < 0.47. Mothers’ substance use severity (*b* = −0.11, *t* = −1.07, *p* < 0.29) and depression (*b* = −0.01, *t* = −0.26, *p* < 0.79) continued not to be predictors, whereas mothers’ trauma symptoms emerged as a significant predictor of frightened–disorganized attachment patterns (*b* = 0.06, *t* = 3.16, *p* < 0.002). Indirect effects were computed for each of 5000 bootstrapped samples. A significant indirect effect of mothers’ ACEs on frightened–disorganized attachment patterns through trauma symptoms was found, *b* = 0.25, BCa CI [0.07, 0.48], representing a large indirect effect and explaining 23% of the variance in frightened–disorganized attachment patterns (i.e., a stark increase from the 9% variance explained by the direct path from mothers’ ACEs to frightened–disorganized attachment patterns). See Table 6 and Figure 1.

#### 3.3.5. Mothers’ Substance Use Severity, Depression, and Trauma Symptoms Predicting Helpless–Disorganized Attachment Patterns

Contrary to our hypotheses, mothers’ substance use severity did not predict helpless–disorganized attachment patterns, *F* (1, 72) = 0.14, *b* = −0.05, *t* = −0.37, *p* < 0.71, *R*^2^ = 0.002. In support of the hypotheses, mothers’ depression, *F* (1, 72) = 14.87, *b* = 0.16, *t* = 3.86, *p* < 0.001, *R*^2^ = 0.17, and trauma symptoms, *F* (1, 72) = 5.88, *b* = 0.05, *t* = 2.43, *p* < 0.02, *R*^2^ = 0.08, positively and significantly predicted helpless–disorganized attachment patterns.

#### 3.3.6. Mothers’ ACEs, Substance Use Severity, Depression, and Trauma Symptoms Predicting Helpless–Disorganized Attachment Patterns

**Figure 2:**
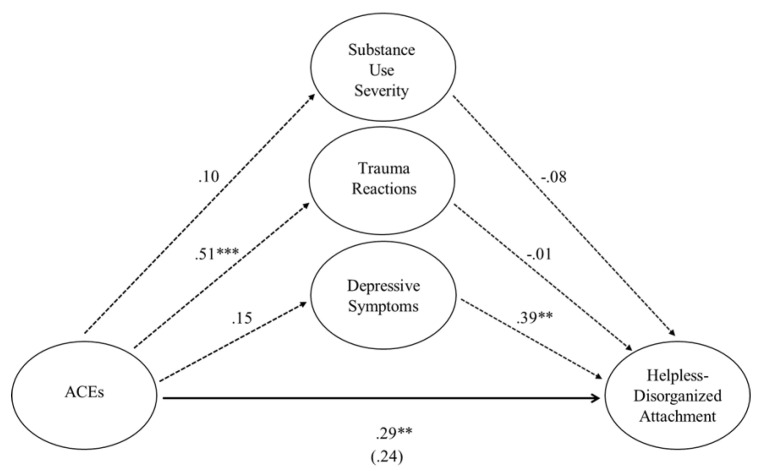
**Standardized regression coefficients for the relationship between mothers’ adverse childhood experiences and helpless–disorganized attachment patterns.***** Note.*** The standardized regression coefficient between mothers’ adverse childhood experiences and helpless–disorganized attachment patterns, controlling for substance use severity and depression and trauma symptoms, is in parentheses. The bold line indicates a direct path, whereas the dotted lines indicate indirect paths. Significance indicated by ** *p* < 0.01 and *** *p* < 0.001.

Collectively, when all predictor variables were entered into the regression equation to predict mother–young child helpless–disorganized attachment patterns, the model was significant, *F* (4, 69) = 5.14, *p* < 0.001, *R*^2^ = 0.23. When mothers’ substance use severity, depression, and trauma symptoms were entered into the equation, mothers’ ACEs decreased in significance but continued to significantly predict helpless–disorganized attachment patterns, *b* = 0.17, *t* = 1.96, *p* < 0.05. Mothers’ substance use severity continued not to predict helpless–disorganized attachment patterns, *b* = −0.09, *t* = −0.76, *p* < 0.45. Mothers’ trauma symptoms also no longer significantly predicted helpless–disorganized attachment patterns, *b* = −0.002, *t* = −0.09, *p* < 0.93. Mothers’ depression emerged as a significant predictor of helpless–disorganized attachment patterns, *b* = 0.15, *t* = 3.28, *p* < 0.002. The significance of this indirect effect was tested using bootstrapping procedures, where indirect effects were computed for each of 5000 bootstrapped samples. Although mothers’ depression explained 23% of the variance in helpless–disorganized attachment patterns (i.e., a stark increase from the 8% variance explained by the direct path from mothers’ ACEs to helpless–disorganized attachment patterns), the indirect effect of depression was not significant, *b* = 0.08, BCa CI [−0.22, 0.19]. See Table 7 and Figure 2.

#### 3.3.7. Second Set of Mediation Analyses: Fathers’ ACEs Predicting Father–Young Child Attachment Patterns

First, the hypothesized path (i.e., direct effect) from fathers’ ACEs to father–young child attachment patterns was examined. In contrast to the hypotheses, fathers’ ACEs did not predict avoidant, *F* (1, 24) = 0.70, *b* = 0.07, *t* = 0.84, *p* < 0.41, *R*^2^ = 0.03; anxious, *F* (1, 24) = 1.12, *b* = 0.09, *t* = 1.06, *p* < 0.30, *R*^2^ = 0.04; frightened–disorganized, *F* (1, 24) = 2.02, *b* = −0.41, *t* = −1.42, *p* < 0.17, *R*^2^ = 0.08; or helpless–disorganized, *F* (1, 24) = 0.91, *b* = −0.29, *t* = −0.95, *p* < 0.35, *R*^2^ = 0.04, attachment patterns with their young children. Thus, the hypothesized direct path was not supported, and the mediational role of fathers’ substance use, depression, and trauma symptoms was not analyzed.

## 4. Conclusions

The objective of this study was to uncover pathways of risk that may perpetuate intergenerational cycles between ACEs and insecure/disorganized attachment patterns in mothers and fathers who are opioid-involved. Historically, much of the literature has considered sequelae stemming from early adversity, including, but not limited to, insecure attachment patterns, lifetime substance use, psychological sequela (e.g., depression and trauma symptoms), and negative parenting [6,12,15,18]. These various risk factors are likely to overlap [6,19,42] and, thus, may be best understood through examination of indirect predictive relationships [24] and collective models that bring these variables together. Given increased concern from the opioid epidemic [3] and evidence that parents who are opioid-involved may demonstrate heightened parenting difficulties [1], this study sought to expand on existing research while also filling gaps in the literature.

Of particular interest, research has focused overwhelmingly on mothers, with fathers seldom being included in empirical discussions of trauma, attachment, and/or parenting [1,25]. Cassidy and colleagues [43] posited that this discrepancy reflected likely challenges in recruiting fathers for research. The challenges of recruiting fathers were compounded in this study by fathers being more likely to be employed than mothers (thereby making them less likely to participate due to work obligations) and by unprecedented difficulties brought on by the COVID-19 pandemic. Nonetheless, this study was still successful in expanding efforts to examine mothers *and* fathers and to explain pathways of risk between parents’ ACEs and insecure/disorganized attachment patterns.

Mediational models examined predictive pathways from mothers and fathers’ ACEs to insecure/disorganized attachment patterns with their young children, with substance use severity, depression, and trauma symptoms as potential mediators. First, the hypothesized path from parents’ ACEs to insecure/disorganized attachment patterns (i.e., direct effect) was supported for mother–young child disorganized attachments *only*, such that mothers with higher ACEs perceived greater frightened–disorganized and helpless–disorganized attachment with their young children. In other words, mothers’ ACEs did not predict mother–young child insecure attachment patterns, only mother–young child disorganized attachment patterns. These findings demonstrated that mothers’ childhood adversity may be related to disorganized attachment patterns in particular, rather than to other insecure attachment patterns in general [44].

The hypothesized pathways (i.e., direct effects) from fathers’ ACEs to father–young child insecure/disorganized attachment patterns were *unsupported* for fathers’ perceptions of all four patterns of attachment. This lack of predictive relationships impeded the ability to learn more regarding the relationship between fathers’ ACEs and father–young child attachment patterns. Despite similarities in our mother and father samples on ACEs, the data highlighted that fathers endorsed higher levels of avoidant attachment patterns than did mothers. No other significant differences were noted in attachment patterns between mothers and fathers. Overall, these findings may imply that mothers and fathers’ perceptions of avoidant attachment patterns may represent different constructs, that mothers’ pathways may be different than those of fathers, and that attachment findings for mothers may not be generalizable to fathers [43]. The sample size for fathers also may have played a role, as more fathers could not be recruited due to their employment responsibilities and the implementation of COVID-19 quarantine protocols in the United States of America during the course of this study. The role of fathers’ attachment consequently necessitates further study [27,43], especially since further exploration of fathers’ indirect mediational pathways could not be completed theoretically in this study.

Contrary to the hypotheses and previous findings [6,7], mothers’ ACEs did not predict their substance use severity, suggesting that a mediational role of substance use severity was not supported. Several theories should be considered in explaining these findings. In this study’s sample, 38% of mothers described prescription opioids (alone or alongside heroin) as their ‘drug of choice’, whereas 50% of mothers described heroin alone as their ‘drug of choice.’ (Opposite percentages were noted for fathers, perhaps explaining some of the differences in findings between mothers and fathers.) Nonetheless, it should be considered that the parents in this sample were receiving medication-assisted treatment at study recruitment. Individuals’ journeys with substance use recovery often vacillate between periods of stability (i.e., sobriety and adherence to treatment) and relapse [27]. It is possible, therefore, that progress in substance treatment and stage of recovery may have buffering effects for mothers’ substance use severity on parent–young child attachment patterns.

In examining the mediational role of mothers’ depression, the hypothesis of this study was not supported. In contrast with this study’s expectations and with previous findings [19,45], mothers’ ACEs did not predict their depression. Interestingly, the majority of mothers (and fathers) in this study exhibited low levels of depression overall, with only 7% of mothers (and 11% of fathers) endorsing severe symptoms. Thus, it may be that this study’s sample was benefiting from the substance use and other treatments that they were receiving. In partial support of hypotheses, mothers’ depression predicted helpless–disorganized attachment. It may be that the helplessness associated with depression also presents itself in the mothering role and interferes with mothers’ abilities to identify young children’s emotional cues and to respond adaptively to them [46]. These data were salient given that disorganized attachment patterns have been associated with the most maladaptive outcomes for young children [47].

When examining the mediational role of mothers’ trauma symptoms, the hypotheses were found to be supported fully. Consistent with previous findings [42], mothers’ ACEs predicted their trauma symptoms, which predicted both frightened–disorganized and helpless–disorganized attachment patterns. When examined collectively, mothers’ ACEs no longer predicted frightened–disorganized attachment patterns. Mothers’ trauma, however, mediated and explained 23% of the variance in the relationship between mothers’ ACEs and frightened–disorganized attachment patterns, representing a large indirect effect as a mechanism of action.

Next, variables were examined collectively in the prediction of helpless–disorganized attachment patterns. In contrast to results for frightened–disorganized attachment patterns, mothers’ ACEs and trauma symptoms no longer predicted helpless–disorganized attachment patterns. Mothers’ depression symptoms emerged as a significant predictor of helpless–disorganized attachment patterns (over and above mothers’ ACEs) and helped to explain 23% of the variance in helpless–disorganized attachment patterns. Bootstrapping procedures, however, were unable to conclude with 95% confidence that mothers’ depression represented a significant indirect effect. Thus, mothers’ depression predicted uniquely helpless–disorganized attachment patterns but did not significantly mediate the relationship between mothers’ ACEs and parent–young child helpless–disorganized attachment patterns.

These data suggested that, rather than mothers’ ACEs being *directly* harmful for mother–young child attachment patterns, the long-term psychological consequences of mothers’ ACEs may carry a greater risk of perceived attachment patterns with their young children. Specifically, mothers’ trauma symptoms acted as a mechanism of action in the relationship between mothers’ ACEs and frightened–disorganized attachment patterns, whereas mothers’ depression predicted uniquely helpless–disorganized attachment patterns above and beyond mothers’ ACEs. These findings corroborated prior research suggesting that mothers’ current psychological symptoms, rather than their histories of childhood adversity, posed the greatest risk to establishing secure bonds with their young children [42]. Disorganized attachment patterns may be exacerbated by the instability and inconsistency characteristic of the opioid recovery process [27]. These findings stressed how mothers’ trauma and depression symptoms, along with their substance involvement, are threats to their young children’s sense of safety and security [48].

The results of this study demonstrated the importance of interventions to be trauma-informed and family-focused when targeting difficulties with parents’ substance use, psychological sequelae, and parent–young child attachment patterns. To date, only one review seemed to comprehensively examine the literature on parenting and opioid use, and fathers who were opioid-involved had been studied seemingly only *once* empirically [1]. Unquestionably, it is vital that future work continues to investigate the specific difficulties and needs exhibited by mothers *and* fathers who have high ACEs and who are opioid-involved to inform evidence-based practice. Future work should utilize a family systems approach to best understand the development of insecure/disorganized attachment patterns between parents and their young children [49]. See Renk and colleagues [50] for a review of evidence-based treatments for parents who are substance-involved. Interventions such as Circle of Security [51], which promote reflection on parents’ own childhood adversity and their current parenting, as well as reflective relationship-based treatments, such as Child–Parent Psychotherapy [52], may also be helpful in mitigating risk for families with young children. Addressing parents’ psychological sequelae through evidence-based intervention approaches may provide further mitigation toward preserving the parenting capacities of mothers and fathers.

The results of this study should be interpreted in the context of its limitations. This study had a relatively homogenous sample, with demographic characteristics potentially compromising external validity. Although lacking in racial diversity, this mostly White/Caucasian sample reflected both the national demographic of opioid users [53] and the racial disparities in those who receive prescription opioids [54] in the United States of America. It should also be noted that this study examined mediations using a cross-sectional design, where ratings of all variables were collected within one time period. Although ACEs were likely to have occurred prior to the other variables of interest (given the nature of this construct), it cannot be determined with certainty whether parents’ depression or trauma symptoms preceded their development of insecure/disorganized attachment patterns with their young children. Further, cross-sectional data do not allow causal claims to be made.

Certainly, a greater sub-sample of fathers would have been optimal. Our efforts resulted in the recruitment of 26 fathers, an ostensibly small sample as compared to the sample of mothers. This sample size was complicated by the fact that fathers more often had to leave for work (relative to mothers) after receiving their medication-assisted treatment, as well as by restrictions put in place for COVID-19. This sample size of fathers was found to exceed that found in previous studies, however [55]. The problematic omission of fathers in attachment research has long been documented [49]. Even after a significant push for more research on father–child attachment, only 16 studies existed on this topic by 2019 [56]. Moreover, only one review could be identified as examining fathers’ opioid use to date [1]. Thus, this study still enhanced the literature on fathers.

This study utilized self-report measures to examine the variables of interest. Although several of the measures included validity scales, the possibility remains that parents may have exhibited positive or negative self-bias [57] in their responses. Nonetheless, there were significant relationships among the variables of interest in this study. Further, Cassidy and colleagues [43] stated the importance of conducting such intergenerational attachment research using self-report measures. It should be noted that, rather than the accuracy of attachment patterns (e.g., on the Strange Situation procedure), this study was interested in mothers and fathers’ perceptions of their attachment patterns with their young children. Nonetheless, future studies would benefit from including young children themselves, especially in conjunction with parent–young child observational paradigms.

In summary, this study uniquely added to the understanding of intergenerational risk pathways between parents’ ACEs and insecure/disorganized attachment patterns with parents who are opioid-involved. The results of this study suggested that mothers’ trauma and depression symptoms play a central role in perpetuating relationships between childhood adversity and disorganized attachment patterns with their young children. As such, these are essential targets for intervention with these mothers. Interestingly, fathers’ ACEs were not predictive of their attachment patterns with their young children. This study recognized that a low sample size of fathers relative to mothers may have impacted relationships among the variables investigated. Further inclusion of fathers in parenting, attachment, and trauma research is vital. Overall, these data highlighted that differences may exist in how mothers and fathers perceive parent–young child attachment patterns. These results have implications for tailoring trauma-informed and attachment-focused parenting interventions to help parents break perpetuating cycles of ACEs and insecure/disorganized attachment patterns.

## Figures and Tables

**Table 1:** Participant demographic information.

Variables	Mothers (N = 75)	Fathers (N = 26)
**Parent’s Age**		
*M* (*SD*)	33.08 (7.79)	34.62 (9.07)
**Child’s Age (Years)**		
*M* (*SD*)	2.83 (1.79)	3.14 (1.61)
**Number of Children**		
*M* (*SD*)	2.16 (1.33)	1.69 (1.09)
**Race/Ethnicity**		
White/Caucasian	80.0%	73.1%
Latino/Hispanic	6.7%	23.1%
Black/African American	8.0%	0%
Multiracial	5.3%	3.8%
**Marital Status**		
Single	70.7%	73.1%
Married	9.3%	15.4%
Divorced/Separated	18.7%	11.5%
**Education Level**		
<High School	6.8%	3.8%
Some High School	15.1%	11.5%
High School Diploma/GED	26.0%	42.3%
Vocational Training	6.8%	3.8%
Some College	42.5%	30.8%
Bachelor’s Degree or Higher	2.8%	7.7%
**Number Living in Home**		
*M* (*SD*)	5.09 (11.15)	3.35 (1.55)
**Substance Use**		
Number of Substances, *M* (*SD*)	5.26 (2.49)	6.46 (2.49)
Opioid Use	37.8%	60.0%
Heroin Use	50.0%	36.0%
**Medication-Assisted Treatment**		
Methadone	82.4%	61.5%
Suboxone	13.5%	26.9%
Vivitrol	4.1%	11.5%
**Religious Affiliation**		
Christian	72.0%	69.2%
**Employment Status**		
Employed	39.7%	84.6%
**Socioeconomic Status**		
<USD 5000	25.0%	3.8%
USD 5000–USD 10,000	6.9%	3.8%
USD 10,000–USD 20,000	19.4%	3.8%
USD 20,000–USD 30,000	13.9%	26.9%
USD 30,000–USD 40,000	15.3%	34.6%
USD 40,000–USD 50,000	9.7%	3.8%
USD 50,000–USD 60,000	2.8%	7.7%
>USD 60,000	7.0%	15.3%

**Table 2:** Descriptive statistics for variables of interest.

	Mothers	Fathers	
**Variables** (Available Range)	*M* (*SD*)	*M* (*SD*)	Actual Range
**ACEs Total Score** (0–10)	3.79 (2.98)	3.00 (2.73)	0–10
**Attachment Patterns**			
Avoidant Attachment (1.00–7.00)	2.38 (0.98)	3.04 (1.09)	1.17–4.44
Anxious Attachment (1.00–7.00)	2.87 (1.09)	3.11 (1.09)	1.00–5.72
Disorganized Attachment (Mother/Child Frightened; 0–30)	9.59 (3.36)	10.88 (4.00)	6–18
Disorganized Attachment (Mother Helpless; 0–30)	10.05 (4.04)	10.65 (4.15)	5–21
**Substance Use**			
InDUC Total (0–50)	36.51 (9.23)	39.58 (8.04)	9–45
M-SAPS Total (0–61)	40.65 (11.17)	46.35 (11.56)	5–60
Parental Substance Use Severity (0–18)	14.16 (3.55)	15.00 (3.83)	2–18
**Depression Symptoms** (0–63)	13.16 (10.33)	14.31 (13.81)	0–47
**Trauma Symptoms** (0–88)	29.35 (24.30)	34.81 (20.90)	0–88

**Table 3:** Independent-samples *t*-tests for mothers’ and fathers’ variables.

	Mothers		Fathers			
	*M*	*SD*	*M*	*SD*	*t*	Cohen’s *d*
Number of Substances Used	5.26	2.49	6.46	2.49	2.12 *	0.48
ACEs	3.79	2.98	3.00	2.73	−1.18	0.28
Avoidant Attachment	2.38	0.98	3.04	1.09	2.89 **	0.64
Anxious Attachment	2.87	1.09	3.11	1.09	0.98	0.22
Frightened Attachment	9.59	3.36	10.88	4.00	1.60	0.35
Helpless Attachment	10.05	4.04	10.65	4.15	0.65	0.15
InDUC Total	36.51	9.23	39.58	8.04	1.51	0.35
M-SAPS Total	40.65	11.17	46.35	11.56	2.22 *	0.50
SUS Total	14.16	3.55	15.00	3.83	1.02	0.23
Depression Symptoms	13.16	10.33	14.31	13.81	0.45	0.09
Trauma Symptoms	29.35	24.30	34.81	20.90	1.02	0.24

***Note*****.** ACEs = adverse childhood experiences; InDUC = Inventory of Drug Use Consequences; M-SAPS = Minnesota Substance Abuse Problems Scale; SUS = parental substance use severity scale. Significance indicated by * *p* < 0.05 and ** *p* < 0.01.

**Table 4:** Correlations among mothers’ ACEs, attachment patterns, substance use severity, depression, and trauma symptoms.

Variables	1	2	3	4	5	6	7	8	9	10
1. ACEs	-									
2. Avoidant Attachment	0.01	-								
3. Anxious Attachment	0.18	0.40 ***	-							
4. Frightened Attachment	0.29 **	0.25 *	0.52 ***	-						
5. Helpless Attachment	0.29 **	0.26 *	0.37 ***	0.46 ***	-					
6. InDUC Total	0.19	−0.10	−0.15	−0.12	−0.12	-				
7. M-SAPS Total	0.10	−0.18	−0.08	−0.06	0.04	0.73 ***	-			
8. SUS Total	0.09	−0.13	−0.05	−0.10	−0.04	0.83 ***	0.83 ***	-		
9. Depression Symptoms	0.15	0.15	0.22	0.16	0.41 ***	−0.01	0.02	0.03	-	
10. Trauma Symptoms	**0** .52 ***	0.15	0.22	0.46***	0.28 *	0.07	0.09	0.01	0.42 ***	-

***Note.*** Significance indicated by * *p* < 0.05, ** *p* < 0.01, and *** *p* < 0.001. Items in bold are significant after Bonferroni correction.

**Table 5:** Correlations among fathers’ ACEs, attachment patterns, substance use severity, depression, and trauma symptoms.

Variables	1	2	3	4	5	6	7	8	9	10
1. ACEs	-									
2. Avoidant Attachment	0.17	-								
3. Anxious Attachment	0.21	0.29	-							
4. Frightened Attachment	−0.29	0.40 *	0.40 *	-						
5. Helpless Attachment	−0.19	0.41 *	0.52 **	**0** .68 ***	-					
6. InDUC Total	0.21	−0.06	0.13	−0.13	0.03	-				
7. M-SAPS Total	0.24	0.08	0.27	0.02	0.19	0.83 ***	-			
8. SUS Total	0.23	0.04	0.18	−0.09	0.07	0.97 ***	**0** .83 ***	-		
9. Depression Symptoms	0.07	0.37	0.25	0.12	0.19	0.01	0.06	0.05	-	
10. Trauma Symptoms	0.23	0.15	0.31	0.02	0.22	0.06	0.21	0.12	0.50 **	-

***Note.*** Significance indicated by * *p* < 0.05, ** *p* < 0.01, and *** *p* < 0.001. Items in bold are significant after Bonferroni correction.

**Table 6:** Mediation effects of mothers’ substance use, depression, and trauma symptoms predicting frightened–disorganized attachment patterns.

		95% BCa CI	
Effect	*b*	Lower	Upper
Total (ACEs; No Mediator)	0.33 **	0.08	0.58
Direct (ACEs)	0.10	−0.18	0.38
Indirect (Substance Use)	−0.01	−0.08	0.03
Indirect (Depression Symptoms)	−0.01	−0.05	0.05
Indirect (Trauma Symptoms)	0.25 **	0.07	0.48

***Note.*** BCa CI = bias-corrected confidence intervals. Items in bold represent significant indirect effects following bootstrapping. Significance indicated by ** *p* < 0.01.

**Table 7:** Mediation effects of mothers’ substance use, depression, and trauma symptoms predicting helpless–disorganized attachment patterns.

		95% BCa CI	
Effect	*b*	Lower	Upper
Total (ACEs; No Mediator)	0.39 **	0.08	0.69
Direct (ACEs)	0.33	−0.01	0.67
Indirect (Substance Use)	−0.01	−0.08	0.04
Indirect (Depression Symptoms)	0.08 **	−0.02	0.24
Indirect (Trauma Symptoms)	−0.01	−0.22	0.19

***Note.*** BCa CI = bias-corrected confidence intervals. Significance indicated by ** *p* < 0.01.

## Data Availability

The data presented in this study are available upon request from the corresponding author due to the privacy of the data and the sensitivity of the sample recruited for this study.
